# Audit of high dose antipsychotics (HDA) prescribing on high dependency unit (HDU) and acute ward, Nepean Mental Health Centre, Nepean Blue Mountains Local Health District, Sydney, Australia

**DOI:** 10.1192/bjo.2021.862

**Published:** 2021-06-18

**Authors:** Adil Jawad, Kristof Mikes-Liu, Christopher Mah, Khyati Roy

**Affiliations:** Nepean Blue Mountains Local Health District

## Abstract

**Aims:**

To measure the rate of patients receiving high dosage antipsychotics.

To review the adherence to maximum recommended doses of antipsychotics as per the product information approved by Australian Therapeutic Goods Administration, product information approved by Medsafe (the New Zealand Medicines and Medical Devices Safety Authority) and Therapeutic Guidelines (Psychotropic Writing Group, 2013)

**Background:**

High dose antipsychotics or combination of antipsychotics are associated with significant adverse effects including QTc prolongation, arrhythmias, sudden cardiac death, seizures, increased incidence and severity of adverse effects, longer hospital stay and possibly increased mortality. High dose antipsychotic prescribing may arise as a result of EITHER single antipsychotic drug prescribed at a daily dose above the recommended limit (High Dose single drug) OR More than one antipsychotic prescribed concurrently where the sum of doses given expressed as a percentage of the SPC maximum of each drug exceeds 100% (High-Dose through the prescribing of multiple drugs).

**Method:**

The data were gathered from all the drug charts for all patients admitted to HDU and Acute ward on 9th April 2019. The Audit standards were 1) Individual antipsychotic dose should be within recommended limit as 100% and 2) Combined antipsychotics should be within recommended limit as 100%

**Result:**

Total number of patients on both the HDU and Acute wards = 33

Total number of patients on antipsychotics = 30

Number of patients on > 100% of recommended cumulative dosage = 13/30 = 43.3%

Number of patients on > 100% maximum limits of regular antipsychotics = 3 = 10%

Number of patients on > 100% maximum limits of PRN antipsychotics = 0/30

Number of patients on 2 antipsychotic = 18/30 = 60%

Number of patients on 3 antipsychotic = 8/30 = 26.6%

Number of patients on 4 antipsychotic = 2/30 = 6.6%

**Conclusion:**

Out of the 30 patients on antipsychotics, almost half were on more than 100% of the recommended cumulative maximum limits of antipsychotics doses, almost 2/3rds were on 2 or more antipsychotic and a quarter on 3 or more. This can be associated with significant adverse effects including QTc prolongation, arrhythmias, sudden cardiac death, seizures, increased incidence and severity of adverse effects, longer hospital stay and possibly increased mortality. There is a need to review PRN antipsychotics prescribed as a norm, clear documentation and need for a protocol for increased vital sign monitoring for patients on high dose antipsychotic treatment.

